# Long-term outcome of percutaneous balloon compression for trigeminal neuralgia patients elder than 80 years: A STROBE-compliant article

**DOI:** 10.1097/MD.0000000000008199

**Published:** 2017-09-29

**Authors:** Xiang Ying, Hao Wang, Shanghua Deng, Yinggao Chen, Jie Zhang, Wenhua Yu

**Affiliations:** a Department of Neurosurgery, Hangzhou First People's Hospital, Zhejiang Chinese Medical University; b Department of Neurosurgery, Hangzhou First People's Hospital, Nanjing Medical University; c Department of Anesthesia and Pain Medicine, Tonglu First People's Hospital, Hangzhou, Zhejiang, P.R. China.

**Keywords:** elderly patients, percutaneous balloon compression, trigeminal neuralgia

## Abstract

This article evaluates the long-term outcome of percutaneous balloon compression (PBC) for trigeminal neuralgia (TN) patients elder than 80 years. A total of 138 elderly patients aged above 80 years with primary TN, who were admitted to Neurosurgery Department, Hangzhou First People's Hospital from January 2007 to December 2011 for PBC treatment, were retrospectively analyzed in this study. The postoperative cure rate of immediate pain was 98.6% (Barrow Neurological Institute [BNI] classes I, II); according to the follow-up, the pain cure rates at 1, 2, 3, 4, and 5 years after surgery were 93.5%, 90.4%, 84.7%, 80.4%, and 72.9%, respectively. In our group, postoperative diplopia was reported in 1 case, masticatory muscle weakness in 3 cases, and herpes labialis in 19 cases. A total of 100% of pain-cured patients exhibited facial numbness and facial hypoesthesia. No serious complications occurred in this group of patients. PBC is an effective and safe procedure for TN treatment and can be employed as the preferred regimen for elderly TN patients aged above 80 years in poorer physical condition.

## 1 Introduction

Trigeminal neuralgia (TN) is a clinically common cranial nerve disease characterized by recurrent, transient, and severe pain in the trigeminal nerve area, which is manifested as electric shocking, knife cutting, and tear-like pain. The pain of TN lasts from several seconds to tens of seconds, and the interval is completely normal. An attack of pain is usually evoked by facial movement, chewing, brushing teeth and washing the face, or by touching the face of a region. The prevalence of TN is estimated to be 182/10 million, and the annual incidence is 3 to 5/10 million.^[1]^ TN occurs frequently in adults and the elderly, and the incidence increases with age.^[2,3]^ The treatment of TN mainly includes drug therapy, radio-frequency thermocoagulation and microsphere compression, percutaneous balloon compression (PBC), stereotaxic radiosurgery, and microvascular decompression (MVD). Currently, medical treatment requires long-term medication to control or alleviate pain. However, about 30% to 75% of patients eventually have to take surgical treatment due to poor analgesic effect or adverse effects after long-term medication.^[4]^ MVD is the best treatment and the longest duration of remission for the treatment of TN by craniotomy to separate the blood vessel from the compressed trigeminal nerve. Nevertheless, the risk of surgery and anesthesia for elderly patients undergoing craniotomy with MVD are still controversial. Many surgeons prefer older patients to select a more minimally invasive approach.

In 1983, Mullan and Lichtor^[5]^ invented PBC through modifying the craniotomy nerve compression created by Shelden and Taarnhoj.^[6]^ There is no requirement for craniotomy during PBC and it thus largely avoids the mortality and complications of craniotomy. Although PBC does not treat TN directing at the etiology like MVD, initial studies have shown that PBC is more successful, safe, and less prone to relapse, becoming more and more popular among older patients. The present study aimed to evaluate the long-term outcome of PBC in the treatment of elderly patients over 80 years of age with TN.

## 2 Materials and methods

### 2.1 Subjects

A total of 138 elderly patients aged above 80 years with primary TN, who were admitted to Neurosurgery Department, Hangzhou First People's Hospital from January 2007 to December 2011 for PBC treatment, were retrospectively analyzed in this study. The study protocol was approved by the institutional review board of Hangzhou First People's Hospital. All patients received informed consent before the procedure and were willing to participate in this clinical study. These 138 patients included 65 males and 73 females with an average age of 86.59 years ± 4.11. Fifty-three patients had pain on the left side and 85 on the right side. Their disease course ranged from 3 to 20 years with the average of 7.2 ± 8.1 years; pain involving branch I was observed in 5 cases, branch II in 37 cases, branch III in 35 cases, branches II and III in 45 cases, branches I and II in 10 cases, and branches I, II, and III in 6 cases; according to the anesthesia risk classification by American Society of Anesthesiologists (ASA), patients of classes I, II, and III were 19, 95, and 24 cases and accounted for 13.77%, 68.84%, and 17.39%, respectively (Table 1). All patients underwent basal 3D computed tomography reconstruction before surgery to observe their oval foramen morphology.

**Table 1:**
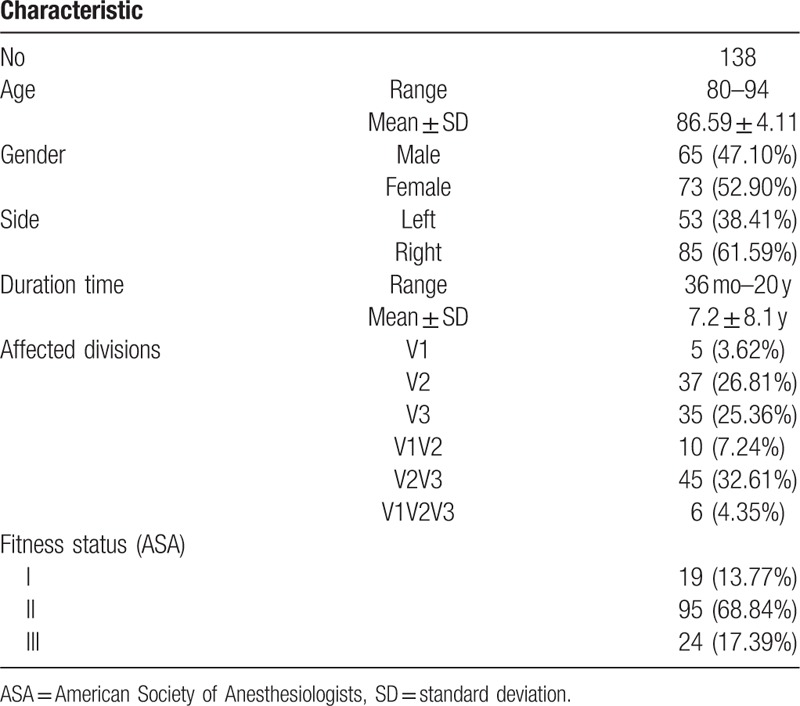
Patients characteristics.

### 2.2 Surgical procedure

**Figure 1:**
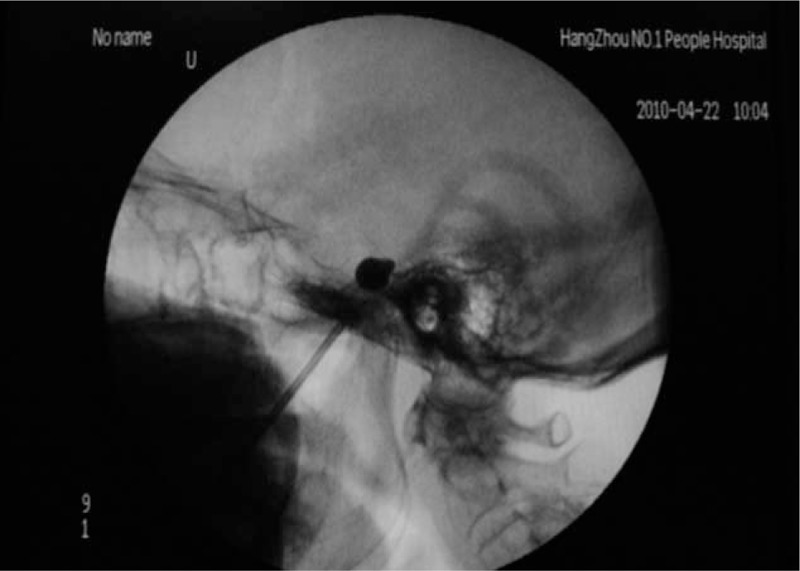
The filled balloon exhibited a pear-like shape as shown by lateral X-ray fluoroscopy.

The surgery was performed under general anesthesia, and the laryngeal mask airway was applied instead of tracheal intubation to reduce patient discomfort under anesthesia; when the patient was placed in the position with tilted head, the percutaneous puncture was performed using 14G rounded mandarin, which was inserted slightly above 1 cm from the orifice lateral angle via the thin wall at the blunt edge under X-ray fluoroscopy, and the puncture needle was placed at rather than beyond the oval foramen; traveling through the puncture needle, 4# Forgarty balloon catheter was put into the Meckel cave via the oval foramen to 1 cm beyond the needle tip and at about 10 to 22 mm from the oval foramen; after that, approximately 0.7 mL (typically 0.5–1 mL) of nonionic X-ray contrast agent iohexol was injected to the balloon; the puncture was successful when the filled balloon exhibited a pear-like shape as shown by lateral X-ray fluoroscopy (Fig. 1). After puncture completion, the balloon compression time usually lasted for 3 minutes, followed by extracting the contrast agent; the puncture point was pressed with a finger for minutes after needle withdrawal.

If intraoperative heart rate and blood pressure monitoring indicated significantly reduced heart rate or other trigeminal depressor responses, the puncture or compression could be suspended and the anesthesiologist could administer atropine and sodium nitroprusside to alleviate the responses. After doing this, the heart rate would quickly return to normal.

### 2.3 Efficacy evaluation

The postoperative follow-up was completed by nonsurgical doctors monthly for consecutive 60 months via telephone, text message, outpatient service, or mail. The postoperative facial pain was measured by Barrow Neurological Institute (BNI) Pain Intensity Score,^[7]^ where class I was defined as completely relieved pain requiring no drug control, class II as partially relieved pain requiring low-dose drug control, class III as partially relieved pain that could be controlled by drugs, class IV as partially relieved pain that could not be controlled by drugs, and class V as unrelieved pain. Facial numbness was determined using BNI facial numbness score,^[8]^ where class I was defined as no numbness, class II as moderate numbness that exerted no impact on daily life, class III as numbness that exerted an impact on daily life, and class IV as numbness that exerted a serious impact on daily life. The aggravation from BNI Pain Intensity Score classes I and II to classes III, IV, and V was regarded as recurrence. IBM SPSS Statistics Version 19 package was employed for statistical processing, and Kaplan–Meier method was used for survival analysis in terms of postoperative pain.

## 3 Results

Table 1 shows the basic information of 138 patients in our group, including age, pain site, course length, etc. These subjects were followed up for an average of 46.83 months (ranging from 6 to 60 months), where 29 cases died during follow-up and 8 cases were lost to follow-up.

**Figure 2:**
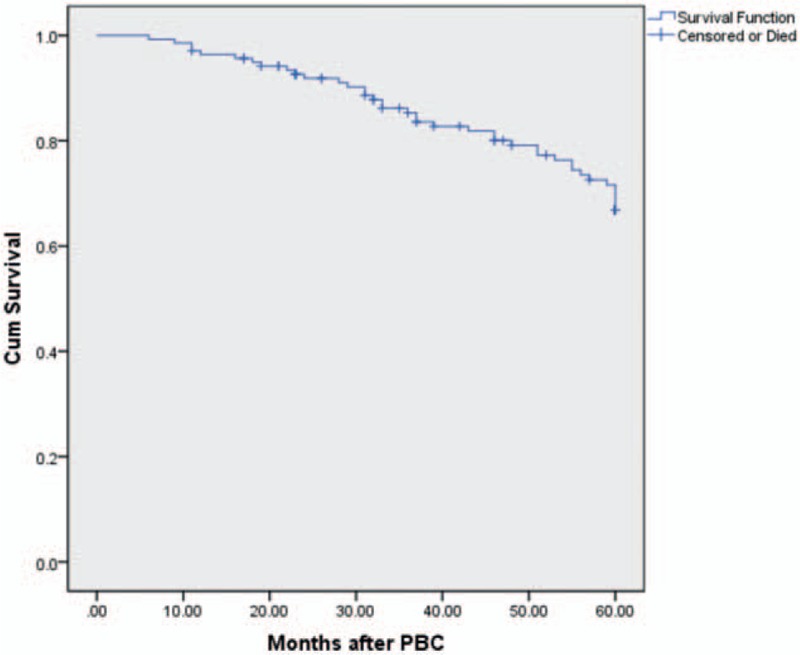
Kaplan–Meier curve comparing the long-term outcomes of patients pain-free off medications after percutaneous balloon compression (PBC).

Of these 138 patients, 1 case failed to complete the path design of straight puncture due to oval foramen deformity, whereas all remaining 137 cases underwent successful puncture upon the first attempt; after the injection of contrast agent, C-arm X-ray device under lateral view showed that the balloon was located near by the posterior clinoid process and sella turcica and filled in a satisfactory pear-like shape with nipple convex to the posterior cranial fossa; the puncture success rate was 99.3%. A total of 112 cases reported trigeminal depressor response manifested by heart rate decline with the incidence of 81.2%, and their heart rate was slowed by 23 beats/minute in average; of them, 1 case developed transient cardiac arrest with the incidence of 0.72%. The postoperative cure rate of immediate pain was 98.6% (BNI classes I, II); according to the follow-up, the pain cure rates at 1, 2, 3, 4, and 5 years after surgery were 93.5%, 90.4%, 84.7%, 80.4%, and 72.9%, respectively (Fig. 2). In our group, postoperative diplopia was reported in 1 case, masticatory muscle weakness in 3 cases, and herpes labialis in 19 cases. A total of 100% of pain-cured patients exhibited facial numbness and facial hypoesthesia, including 121 cases of facial numbness BNI II and 15 cases of facial numbness BNI III, which accounted for 89% and 11%, respectively. No facial numbness or facial hypoesthesia was observed in 2 cases reporting ineffective treatment. No serious complications occurred in this group of patients.

## 4 Discussion

TN is a common and frequently occurring disease. Comprehensive treatment measures must be applied due to the complex pathogenesis of TN. Treatment options include drug therapy, percutaneous therapy, and MVD. For newly diagnosed TN patients, cranial computed tomography/MR should be examined first to exclude secondary TN, and secondary TN should be treated according to the cause of secondary TN. If it is a primary TN, medication should be given firstly, such as carbamazepine, starting from a small dose until the maintenance dose, meanwhile, regular liver and kidney function, as well as blood routine test should be applied during treatment. Surgical treatment is recommended if the patient is unable to tolerate side effects of the drug or is otherwise ineffective.

With the improvement of modern microsurgical techniques, the accumulation of physician experience has been reported in MVD, and satisfactory results have been achieved in the treatment of elderly patients with TN. However, due to the existence of many chronic diseases in the elderly patients, such as diabetes, heart disease, and hypertension, the incidence of cardiovascular and cerebrovascular accidents is still relatively higher, the risk of perioperative and anesthesia is higher accordingly; therefore, we still need to be conservative about the implementation of the operation. How to choose a safe and effective surgical treatment to rid elderly patients from pain and improve their quality of life is our further discussion and research topic. In the study, PBC provides us with a safe and effective method that is featured by simple technology, short operation time, and high safety. Besides, the procedure will be performed under general anesthesia without patients’ coordination, and the treatment process is basically painless. In addition, it avoids the limitation of craniotomy for the patient's age, basic diseases, and poor health. Therefore, for elderly patients with generally good physical condition, normal cardiopulmonary function, and definite vascular compression by MRA, MVD, or PBC can be performed according to the attitudes of patients. On the other hand, PBC can be recommended if the patient is old and weak with poor general condition.

As one percutaneous puncture minimally invasive technique, PBC is internationally used for the treatment of primary TN in recent years. Its principle may be associated with: the selective damage to touch-conducting large-caliber myelinated fibers after the balloon compression to the semilunar ganglion, which blocks the TN conduction pathway and simultaneously suppresses the trigger inducing pain attack; or relief of the potential local nerve compression in trigeminal nerve.^[9]^ By compressing rabbit trigeminal ganglion, Brown and Hoeflinge^[10]^ found demyelinating changes in the large myelinated fibers of trigeminal nerve and thus speculated that the treatment of TN with balloon compression was associated with its selective effect on large myelinated fibers. This approach avoids craniotomy-related inherent risk to a great extent and provides a superior treatment option for patients with advanced age and ASA II or above.

Balloon shape, location, internal volume as well as pressure compression time are several major technical parameters affecting PBC surgical outcomes.

Almost all authors hold that the sign of successful surgery is the “pear-like” balloon with nipple convex to the foramen trigeminale as shown by lateral X-ray; pear-shaped balloon indicates the consistency between balloon volume and Meckel cave,^[11]^ and the pear-shaped tip located at the back of semilunar ganglion can thereby constitute a compression against trigeminal semilunar ganglion. Asplund et al^[12]^ divided the balloon location into low type, medial type, and high type according to the distance between balloon upper edge and the sella turcica. The location with the upper edge at more than 0.2 cm to the sella turcica was defined as the low type, that at less than 0.2 cm as medial type, whereas that hardly outside the sella turcica as high type. All 3 types of balloon position above can achieve surgical effect, but excessively high location or excessively large balloon volume may involve the abducens and further cause diplopia.

The study by Lee and Chen^[13]^ demonstrated that different location of microballoon in the Meckel cave could produce different pressure even if the microballoon volume was identical. According to their measurements, the average pressure in the Meckel cave, posterior cranial fossa, and outside the oval foramen was 393, 282, and 320 kPa, respectively; Lobato proposed a pressure range of 980 to 2080 mm Hg with the average of 1200 mm Hg and suggested that the balloon might fail to effectively compress the trigeminal semilunar ganglion if the intraballoon pressure was less than 600 mm Hg. By reducing their initial compression time of 3–7 to 1–2 minutes, Fraioli et al^[14]^ found no decline in surgery success rate but significantly decreased complications. Lee and Chen^[13]^ also noticed that microballoon compression for 60 seconds significantly reduced the complication of postoperative facial hypoesthesia in patients suffering pain involving trigeminal nerve branch I. Many scholars believe that there is no significant difference in efficacy for balloon compression ranging from 1 to 3 minutes, but compression of greater than 3 minutes may increase the incidence of postoperative facial discomfort. In the treatment of TN with PBC, it is of great importance to reduce the postoperative complications as well. However, universal standards on balloon compression time have not been established in existing literature, and surgeons have to perform a comprehensive analysis based on patient's ethnicity, age, underlying diseases, pain degree, pain course, and skull base anatomic structure. In order to lower complications through reducing balloon compression time without causing decrease in efficacy, the compression time in our group was about 3 minutes, and no obvious drawbacks were observed.

Through reviewing literature reports, Meglio et al^[15]^ found that the remission rate of PBC, MVD, and RF thermocoagulation was similar in the treatment of early pain (79%–95.6%, 80%–100%, and 81.8%–95%, respectively). Montano et al^[16]^ carried out a similar literature study and suggested good early remission rate in patients undergoing different therapeutic procedures, which showed no significant intergroup difference; the efficacy of PBC and MVD was equivalent in terms of the long-term pain remission rate. Providing varying evaluation criteria for surgical efficacy between different authors and considerable heterogeneity between different literature regarding the description of long-term follow-up results and the follow-up duration, the quality of evidence provided by meta-analysis is poor. Therefore, qualified multicenter randomized controlled trials designed to further compare the long-term efficacy between PBC and other surgical procedures may offer firmer evidence-based medical basis for the treatment of TN with PBC.

Sudden rise in blood pressure, tachycardia, bradycardia, sudden drop in blood pressure, and other hemodynamic changes appear to be the common intraoperative complications. Chowdhury et al^[17]^ named such conditions as trigeminal cardiac reflexes, whose specific mechanism remains unclear; posing a potential life risk to patients undergoing surgery. Trigeminal cardiac reflexes is a common phenomenon in the process of PBC for the treatment of TN, which generally does not affect the treatment process by taking appropriate preventive and therapeutic measures. Surgeons and anesthesiologists must be fully aware of this possible cardiac suppression response, and corresponding preparations must be made for the surgical and pharmaceutical aspects. In the treatment of TN, the common complications of PBC are relatively mild, including hypesthesia, dysesthesia or even hyperesthesia in the trigeminal innervating areas, masticatory muscle weakness, corneal hypoesthesia, and loss of corneal reflex, as well as viral herpes. Other complications, such as aseptic meningitis, oculomotor nerve and/or trochlear nerve palsy, and elevated odor threshold, are relatively less encountered.^[18–20]^ By summarizing the postoperative complications of PBC in 100 patients, Lichtor and Mullan^[21]^ concluded that hypesthesia, numbness, and even hyperesthesia in the trigeminal innervating areas were the most important complications, whose severity was associated with the duration of pain relief; of these 100 cases, hypesthesia was reported in a majority of patients with dysesthesia in a minority, while hyperesthesia was observed in 4 cases only.

## 5 Conclusion

PBC is an effective and safe procedure for TN treatment and can be employed as the preferred regimen for elderly TN patients aged above 80 years in poorer physical condition.
